# Similarity searches in genome-wide numerical data sets

**DOI:** 10.1186/1745-6150-1-13

**Published:** 2006-05-30

**Authors:** Galina Glazko, Michael Coleman, Arcady Mushegian

**Affiliations:** 1 Stowers Institute for Medical Research, 1000 E 50^th ^St., Kansas City MO 64110, USA; 2 University of Rochester Medical Center, Rochester, NY 14642, USA; 3 Department of Microbiology, Molecular Genetics, and Immunology, University of Kansas Medical Center, Kansas City, KS 66160, USA

## Abstract

We present psi-square, a program for searching the space of gene vectors. The program starts with a gene vector, i.e., the set of measurements associated with a gene, and finds similar vectors, derives a probabilistic model of these vectors, then repeats search using this model as a query, and continues to update the model and search again, until convergence. When applied to three different pathway-discovery problems, psi-square was generally more sensitive and sometimes more specific than the ad hoc methods developed for solving each of these problems before.

**Reviewers:**

This article was reviewed by King Jordan, Mikhail Gelfand, Nicolas Galtier and Sarah Teichmann.

## Open peer review

Reviewed by by King Jordan, Mikhail Gelfand, Nicolas Galtier and Sarah Teichmann. For the full reviews, please go to the Reviewers' comments section.

## Background

Genome era produces large multidimensional datasets, which need to be analyzed in robust, quantitative ways. The first-aid response to the advent of gene expression data and other genome-scale measurements was cluster analysis. The techniques of global partitioning of the data, such as K-means, partitioning around medoids, various flavors of hierarchical clustering, and self-organized maps [1-4], have provided the initial picture of similarity in the gene expression profiles, and helped to infer functional links between genes. However, cluster analysis has its drawbacks. Typically, once a gene is assigned to a cluster, it remains in that cluster, even though many genes participate in more than one pathway. Furthermore, the degree of intra-cluster similarity between expression profiles may not be the same for every set of functionally linked genes; this puts limitations on the use of cutoffs and on the number of clusters that can be predicted with confidence. Several approaches have been suggested to overcome these problems, for example, iterative clustering and iterative maximization of the partition quality [5].

Another approach to finding functionally relevant groups of genes is network derivation, which has been popular in the analysis of gene-gene and protein-protein interactions [6-10], and is also applicable to gene expression analysis [11,12]. This class of methods overcomes the inflexibility of hierarchical clustering/partitioning approaches. However, network definition is also confronted with the issue of estimating statistical significance, and, as with partitioning approaches, the significance threshold can be different in different parts of the same network [13]. In addition, visualization and navigation of links in the highly connected network poses its own set of computational challenges.

Although the general picture of dependencies between genes and their products can be obtained by these methods, in fact many biological questions asked of the genome-wide measurements have little to do with global clustering or with laying out the whole network. Rather, a commonly encountered task is to discover the neighbors of a point, which represents a set of measurements associated with a gene or a protein. Finding such groups does not require the knowledge of all genome-wide correlations – the fundamental task here is to discover and rank similarities that are local with regards to the complete measurement space.

Pathway reconstruction and discovery of functional links can be cast as tasks belonging to this class. We are given one or a few members of a pathway, and would like to infer the other, functionally linked members of the same pathway. Functional links may be discovered, for example, by similarity of expression profiles [2,14,15], or by similarity between the set of protein-protein interaction partners [16,17], or by co-inheritance of groups of genes across different genomes [18]. Assuming that the query belongs to a functionally and evolutionarily defined module, we want to find as many members of this module as possible. At the same time, many – perhaps most – entities in the measurement space are not involved in the module of our interest and, with correctly chosen statistics, should display only the random-level similarity to the query.

This logic has been exploited for decades, and with considerable success, in another area of computational biology, i.e., in sequence similarity-based prediction of biopolymer structures, functions, and evolutionary origins. The standard first step in studying new sequence is a database search, performed by a program like BLAST [19] or PSI-BLAST [20]. If there is a similarity between an uncharacterized query sequence and a better-studied sequence in the database, this information can be used for structural, functional, and evolutionary inferences. At the same time, the similarities between sequences that are unrelated to the query are not of interest, and there is often no need to examine them at all.

In this work, we apply similar logic to searching the multidimensional space of genome-wide numeric datasets. The search is performed by an iterative pattern-matching program that was inspired by PSI-BLAST, and is called psi-square ("pseudo-PSI"). The idea of the algorithm is to start with a numerical pattern of interest (gene expression profile, gene occurrence pattern, protein interaction list, or any other), to find group of highly similar patterns, to derive a probabilistic model of that group, and to repeat database search using this model as a query. In the rest of this paper, we describe the psi-square algorithm and software, and apply it to three pathway-discovery problems, which make use of very different types of genome-wide datasets.

## Results

### Algorithm

The summaries of genome-wide measurements associated with a given gene have been called "profiles" and "patterns" (e.g., "phyletic patterns" [21,22] or "expression profiles" [15]). For the sake of generality, we will call a set of numbers (measurements) associated with the *i*^th ^gene "a gene vector". In different experiments, the same gene can be associated with a phyletic gene vector, an expression gene vector, a protein-protein interaction gene vector, etc. Different measurements for the same gene can, in principle, be combined. In this study, however, we are concerned with the cases when each coordinate of each vector represents one and the same type of measurement.

A gene vector space, or vector database, is a set of *M *gene vectors *X*_*i*_, of dimensionality *N *each, where *N *is the number of data points/experimental conditions associated with each gene. We assume that a vector of interest, called "query", is known (either produced by actual measurements, or made up), and we want to find similar vectors in the database. The query may represent a set of relative or absolute measurements, as with gene expression data; or it may consist of numerically encoded discrete states, such as gene presence-absence, gene expression or lack thereof; or it can be a probabilistic model derived from a series of related vectors. We will use "profile" to refer to the set of all probabilities associated with every coordinate in a vector [23] and will use "dimension-specific scoring matrix" (DSSM) as a synonym for profile.

The psi-square algorithm searches the database for vectors that are similar to the query vector (or query profile/DSSM, i.e., a probabilistic model of several related vectors). The program takes query vector *X*_*i *_as its input and produces a set of similar gene vectors (a subset of the vector database) as its output. The logic of the algorithm is reminiscent of iterative sequence similarity search and has two iterative steps: (1) compare the profile formed from the query vector and, perhaps, other closely related vectors, to the entire vector database; (2) update the profile based on the high-scoring matches, producing the DSSM of scores *s*_*kj*_, where *k = 1,..,K and j = 1,..N*. There are two user-defined parameters for similarity thresholds (*r *and *s*) governing the profile update and *K *is an additional parameter that corresponds to the number of discrete categories (see Materials and Methods for more detail).

Vector dimensionality (*N*) may correspond to the number of different treatments or time points in gene expression experiments, genomes in the phyletic pattern space, etc. A vector or a vector set of interest are called target vector set, *T*, and the complete vector database is called background vector set *B*. Every element *s*_*kj *_of the matrix is the log-odds ratio *s*_*kj *_= log{Pr(*a*_*k*_*,cj|T*)*/*Pr(*a*_*k*_*,c*_*j*_*|B*)}, where Pr(*a*_*k*_*,c*_*j*_*|T*) is the probability of observing the value *a*_*k *_at coordinate *c*_*j *_in *T*, and Pr(*a*_*i*_*,c*_*j*_|*B*) is the probability of observing *a*_*k *_at the same coordinate in *B*. The probability is estimated as the frequency (*f*^*T*^_*kj *_or *f*^*B*^_*kj*_) of the given observation at the specific coordinate in the target or background vector sets, respectively. This scoring scheme is familiar from the theory of sequence comparison. In the context of sequence similarity searches, the log-odds scores derived from the target dataset are known to be optimal for signal recovery [24]. High sensitivity of this scoring scheme in our hands (see below) suggests that log-odds scores may be likewise close to optimal when applied to different types of gene vectors, though this proposition remains to be formally proven.

### Phyletic vectors (problem 1)

Information about phyletic distribution of orthologous genes, i.e., presence and absence of orthologs in completely sequenced genomes, is of interest, because functionally linked proteins tend to be co-inherited in the same subsets of genomes [25]. Informally, co-inheritance has been approximated by low Hamming distance (e.g, three bits or less) between phyletic vectors [18], but a more systematic analysis indicated that other distance/similarity measures, in particular those based on correlation, can greatly improve the sensitivity of functional inference from co-inheritance [26]. One case study in this work is the search for new proteins associated with functioning of bacterial flagellae, based on their co-inheritance with the known flagellar components.

**Figure 1:**
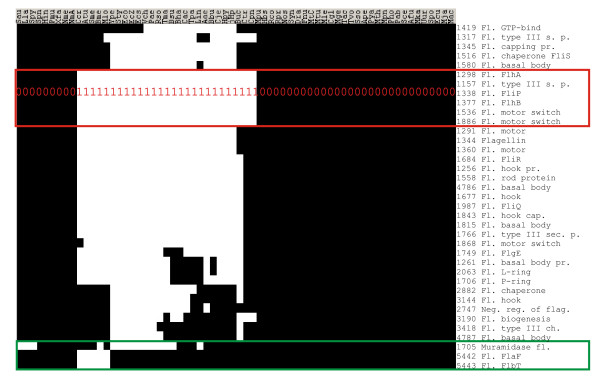
Phyletic vectors of 37 bacterial COGs related to flagella biogenesis and function. Bacteria with flagellar phenotype and flagella-related genes are clustered to highlight the 'flagella genomic signature'.

Flagellae, the sensory and locomotive organs, are found in 23 bacteria out of 50 in the COG database. Four bacteria in the database, *Chlamidia trachomatis, Chlamidophila pneumoniae, Buchnera sp. APS*, and *Yersinia pestis*, do not have flagellae, but contain several genes orthologous to flagella assembly factors in other species, presumably because these genes have additional functions, such as assembly of other extracellular protein complexes [27,28]. The genomic signature of the flagellar biosynthetic and structural genes is represented by a vector with 27 coordinates set to one and 23 coordinates set to zero (Figure 1). There are only 6 COGs characterized by such a phyletic vector, yet at least 37 bacterial COGs are known or inferred to be directly involved in bacterial flagella biogenesis and function (Figure 1). Thus, phyletic vectors of at least 31 flagella-related genes mismatch the query constructed on the basis of flagellar phenotype – in the extreme case, by 22 points (Figure 1). These mismatches are due, in part, to the modularity within the flagella apparatus, where some genes function autonomously and are inherited independently, as in the aforementioned aflagellate bacteria. Differences between flagella-related phyletic vectors may be also explained by differential gene losses and functional takeovers by unrelated genes [27,28]. Regardless of the reason for patchy distribution of flagellar components, many of them can not be sensitively and specifically discovered by exact matching to a made-up genomic signature, nor with naïve methods of Hamming distance-based matching.

Levesque et al. [29] have suggested a series of algorithms that make functional predictions on the basis of phyletic vectors and set theory. This "Trait to Gene" software (TTG in the sequel) identifies 33 COGs as associated with flagella phenotype at the most sensitive similarity threshold 0.65 (Figure 1 and see ref. [29] for details of selecting the threshold value). Among those, 27 COGs have annotations indicating their involvement in flagella. Thus, the approach results in at least 82% true positives and recovered 73% of the 37 known flagellar COGs (Table 1).

**Table 1:** Sensitivity and specificity of psi-square, TTG and PP algorithms in prediction of flagellae components.

	Psi-square: single query	Psi-square:combined query	TTG	PP
False Positives (FP)	16	39	6	24
True Positives (TP)	29	34	27	22
False Negatives (FN)	8	3	10	15

**SPECIFICITY**	**0.644**	**0.466**	**0.818**	**0.478**
**SENSITIVITY**	**0.784**	**0.919**	**0.730**	**0.595**

Number of predicted proteins:	45	73	33	46

Another approach for functional prediction from phenotype has been suggested by Jim et al. [30]. Their method computes the phenotype propensity (PP), i.e., the ratio of two numbers, the frequency of the genomes that have both phenotype and protein of interest, and the frequency of all genomes which have the same protein, whether or not they also have the phenotype of interest. Proteins that appear only in genomes with given phenotype have the highest propensities. In the case of flagella phenotype, the PP approach identifies 46 highest-propensity COGs, corresponding to 60 *E.coli *proteins. Twenty-two of them (59%) overlap with 37 known flagellar COGs (Table 1, and see figure in additional data file 1).

**Figure 2:**
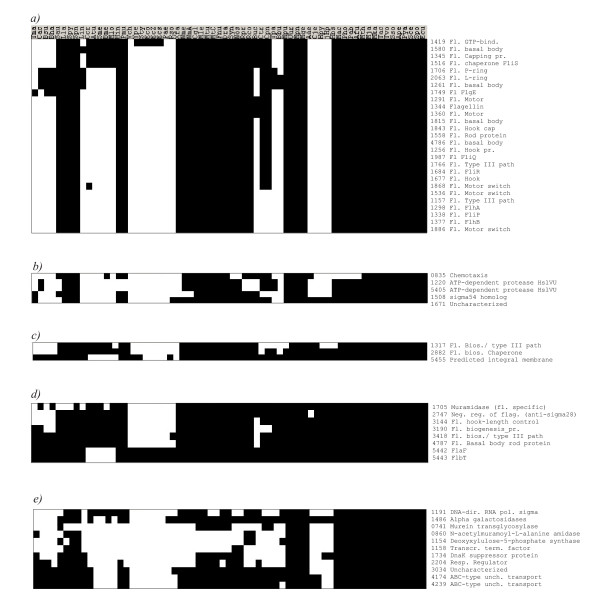
Phyletic vectors and COGs associated with flagella phenotype, identified by psi-square and TTG algorithms (45 COGs and 33 COGs, respectively), with COG1298 used as a query. a) 27 COGs in benchmark (see text), also found by psi-square and TTG; b) 5 COGs found by psi-square and TTG; c) 2 COGs found by psi-square and in benchmark and one COG found by TTG only; d) 8 COGs found in benchmark only; e) 11 COGs found by psi-square only. COG numbers and functional annotations are shown in the right-hand column. Note that the species' order in this figure is different from Figure 1 and reflects the evolutionary relatedness of species.

We applied the psi-square algorithm to find vectors most similar to the flagella genomic signature vector (Figure 1). COG1298, one of the six COGs perfectly matching the flagellate phenotype, was used as a query, with *r *set at 0.6. In this search we recovered 45 COGs at the first iteration. Twenty-nine of these COGs were involved in flagella assembly or function (this corresponds to 78% of all known flagellar proteins). Thus, the naïve psi-square approach had higher sensitivity, but lower specificity, than TTG (Figure 2, Table 1), and exceeded the PP approach in both specificity and sensitivity (Table 1).

To supplement the naïve psi-square search, we collected 29 flagella-associated COGs found at the first step of the analysis and used them as queries in further rounds of psi-square searches, with the *r *parameter set more conservatively at 0.7. The union of all newly found matches gives 73 vectors, with 34 true positives, i.e. 92% of the known flagellar COGs (figure in additional data file 2). Seven flagellar components were predicted by psi-square at this step, but were missed by TTG (figure in additional data file 2), indicating higher sensitivity of psi-square towards these outlying vectors (Figure 1).

Thirty-four COGs were predicted by psi-square only (figure in additional data file 2). Phyletic patterns of these COGs were much "patchier" than the flagella genomic signature (Figure 2e, and figure in additional data file 2). At least five of the proteins found only by psi-square, COG2160, COG2230, COG2356, COG0854, COG3154, appear to be unrelated to flagella function and biogenesis (figure C in additional data file 2). On the other hand, among the 34 genes uniquely identified by psi-square, nine are involved in cell division, shape determination, and chemotaxis. These are most likely not spurious matches, as the recent evidence suggests several linkages between these processes and flagellar function [31,32]. We expect that several of the remaining COGs, for example some of the transcriptional regulators (COG1221, COG3829, COG3835) and signal transduction proteins (COG3852, COG3605) are also involved in the regulation of flagellar biogenesis. Moreover, 3 proteins found by psi-square (COG1699, COG2257, COG3034, figure in additional data file 3) may have previously unreported connections to flagellar phenotype, based on contextual information from STRING database (Table 1 in additional data file 3).

In sequence similarity searches that employ PSSMs and other probabilistic models, the result may be biased by zero frequency of some states in the query and in the first few relatives included in the model. Several ways to regularize the PSSM, including pseudocounts, Dirichlet mixtures, and Bayesian approaches, have been proposed [33-35]. We tested the effect of incorporating pseudocounts into DSSM, using the ratio of column diversities [36]. In that case, use of pseudocounts led to more false-positives than the simple odds ratio (384 phyletic vectors were found instead of the original 73). This result is similar to what was reported by Schaffer et al. [37] in the case of sequence data. Therefore, we decided not to pursue the regularization strategies in Problems 2 and 3.

### Gene expression vectors (problem 2)

The lifecycle of the malaria parasite includes three stages: the mosquito, liver and blood stages. The blood stage is responsible for all of the malaria symptoms and mortality in humans and is therefore an important target for vaccine development [38]. Despite much research and development, an effective malaria vaccine is still unavailable [39]. Recently, the transcriptional program of the asexual intraerythrocytic development cycle (IDC) of *P.falciparum *has been characterized [40]. The parasite-specific genes, especially those related to the initiation of the IDC (merozoite invasion), may be good candidates for vaccine development.

Several candidate antigens have been identified *in P.falciparum*. Most of them are expressed on the parasite cell surface, in particular within apical organelles involved in merozoite invasion [38]. Among the best-studied invasion proteins are seven malaria vaccine candidates, AMA1, MSP1, MSP3, MSP5, EBA175, RAP1 and RESA1. Their expression profiles undergo sharp induction during the mid-to late schizont stage. In order to find additional vaccine candidates, Bozdech et al. [40] compared the Euclidean distances between expression profiles of seven antigens and the rest of plasmodium transcriptome, and the 5% of this distribution with the lowest distance (5%ED) was proposed as a plausible set of vaccine candidates. The 5%ED set of 262 ORFs included virtually all known merozoite-associated genes.

**Figure 3:**
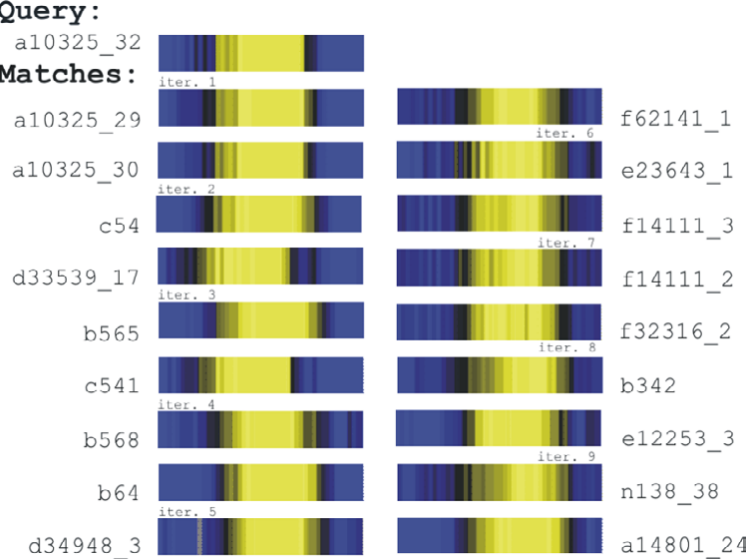
Expression vectors for the closest matches retrieved by psi-square with query PFA0110w in Plasmodium IDC dataset. Two best matches per iteration (nine iterations before convergence) are shown.

We used the psi-square approach to find proteins involved in merozoite invasion in the IDC set. Seven independent searches were initiated with seven antigens as queries. (When one ORF was represented by multiple probes on the chip, we chose the vector with the highest average correlation to the other vectors). We tried several thresholds for correlation and several values of the *K *parameter, with 24 parameter settings altogether (Table 2 in additional data file 3). The correlation threshold 0.9 and K = 15 maximized the number of iterations and new matches. Figure 3 presents matches found during several iterations of psi-square for queryPFA0110w (ring-infected erythrocyte surface antigen precursor). In sum, psi-square and 5%ED identified, respectively, 596 and 419 probes. There were 409 probes found by both approaches, 187 probes corresponding to 151 unique ORFs found only by psi-square, and 10 probes found only by 5%ED.

The average maximum time of expression for 187 unique probes matched 30 hours, i.e., the beginning of the schizont stage. Among them were several already *known P.falciparum *antigenes, such as RESA-H3 (PFB0915w), MSP8 (PFE0120c), octapeptide-repeat (ORA) (PFL0035c), PF70 (PF10_0025), membrane protein ag-1 (PFD0255w), RESA-2 (PF11_0512), tryptophan/threonine-rich antigen (PF08_0003), and transmission-blocking target antigen (PF13_0247). None of these proteins have been identified by the 5%ED method.

In this Problem, we cannot estimate specificity and sensitivity within the previously established framework: unlike the Problem 1, the list of true positives is unknown in this case, and, moreover, we intentionally tuned parameters of psi-square so as to find more candidates. Therefore, to compare biological relevance of two approaches, we examined the sequence properties of the two sets of hypothetical proteins (HP), found either by the psi-square approach only (HP_s1_,108 proteins), or by both the psi-square and 5%ED methods (HP_s12_, 154 proteins). The structural properties of the proteins in these two non-overlapping sets are nearly the same, and at the level of predicted molecular function, the two groups of proteins exhibited many common features (Tables 3 and 4 in additional data file 3). Both sets are depleted of the housekeeping genes involved in genome expression, in intermediate metabolism, and in signal transduction from cytoplasm to the nucleus. Among the proteins with predicted enzymatic activity, there is a clear prevalence of domains involved in lipid biosynthesis and membrane remodeling. Also seen in both sets are proteins with chaperone activity, components of cytoskeleton and of secretory vesicles, and multiple protein kinases and phosphatases (Table 4 in additional data file 3). These observations are compatible with the idea of regulated changes of cell surface and cell shape upon transitioning to the merozoite phase. Interestingly, HP_s1 _and HP_s12 _recover different bona fide antigen-related proteins (RESA in the case of HP_s1 _and AMA-1 and MSP7 in the case of HP_s12_).

These results indicate that psi-square is quite specific towards the putative proteins involved in merozoite invasion. At the same time, psi-square is more sensitive than 5%ED method: psi-square has recovered many ETRAMPs, expressed mostly at early ring stage and located at the parasite-host cell interface, as well proteins identified by MudPIT as parasite proteins on the surface of the infected erythrocyte (PIESPs, Florens et al. [41]), none of which was detected by 5%ED. Psi-square also identified PFE0340c, an ortholog of the rhomboid protease involved in adhesin cleavage during invasion of another apicomplexan parasite, *Toxoplasma gondii *[42]. This suggests an additional strategy of anti-malaria drug development, namely to screen for small-molecule inhibitors of the enzymes involved in membrane remodeling upon merozoite invasion.

### Protein-protein interaction vectors (problem 3)

The majority of cellular processes are carried out by multiprotein complexes [43], and analysis of their composition is of great interest. Screening of protein-protein interaction (PPI) at a large scale can be done with yeast two-hybrid technology [44], which registers only pairwise PPI, and with various affinity purification schemes [45], which record the protein content of a complex but not individual interacting pairs. High-throughput screens are noisy because of non-specific binding, fragmentation into subcomplexes [17], low reproducibility [45] and other factors. True protein complexes must be discerned by a combination of analytical biochemistry and computational techniques [45].

We used the psi-square strategy to identify protein complexes in yeast affinity purification data from Gavin et al. [45]. The PPI vector space can be set up in several ways. For example, purification vectors can be compared in the space of protein coordinates, or else protein vectors can be compared in the purifications' space. In the former case, the search result would be the set of purifications similar to the purification of interest; in the latter case, the result is the set of proteins co-purifying with the query protein.

We applied psi-square to recover the contents of the protein complex responsible for post-transcriptional maturation of the 3'-end of eukaryotic pre-mRNA. This reaction occurs in several steps, including site-specific cleavage, polymerization of the poly(A) tail, and trimming of adenylate residues to mature length [46]. In yeast, the major components of these processes are poly(A)-binding protein (Pab1p), poly(A) nuclease (PAN), and three multidomain complexes, CFIA, CFIB, and CPF [47]. Using Ptal as the first bait, Gavin et al. [45] experimentally identified 12 of the 13 known components of the polyadenylation complex and 7 new putative components.

The psi-square search of interaction vectors initiated with Pta1 converged in one iteration (*r *= 0.6), detecting 10 known components of the polyadenylation machinery (Cft1p, Cft2p, Glc7p, Pap1p, Pfs2p, Pta1p, Ysh1p, Fip1p, Yth1p, Rna14p) and two putative components, Ref2p and YKL059C, which have been also identified by Gavin and co-workers. We then applied the same strategy as with flagella proteins in Problem 1, running 13 psi-square searches, one for each already found component, and taking the union of all newly found vectors. This strategy led to identification of 5 additional components (Ssu72p, YOR179C, C1p1p, Pcf11p, Rna15p) which were also found in TAP-purification analysis [45]. In sum, our analysis identified all components found by TAP, except two, Pab1p and YKL018W (Figure in additional data file 4).

The orthogonal search, initiated with a purification vector of all proteins retrieved when Pta1 was used as a bait, converged at one iteration (*r *= 0.6), resulting in 11 similar purifications. These purifications included 33 proteins (Figures in additional data files 4, 5). Thus, the protein-based query retrieves a set of proteins virtually identical to the original complex found by Gavin et al. [45], whereas the purification-based query discovers many additional proteins. Sequence analysis indicates that among these new findings there are two RNA helicases Has1p and Dbp4p, putative RNA modification enzymes Cbf5p (pseudouridylate synthase-like) and Nop1p (methyltransferase-like), as well as nucleolar proteins Nop56p, Nop58p, and Rsa3p. Many of these proteins are more familiar as components of processosome, the complex that is responsible for maturation of ribosomal RNAs. Recent evidence, however, suggests the existence of extensive cross-talk between processing of rRNA and mRNA [48], and our results point in the same direction.

We also compared our results with hierarchical clustering of the same TAP with parameters set as in Krause et al. [17]. Interestingly, the cluster that included Pta1 also contained the same 33 proteins that were retrieved by psi-square when the Pta1-baited purification was used as a query.

## Discussion

Many clustering approaches tend to underestimate functional relationships among gene vectors [49-51]. Our approach addresses some of the limitations of global clustering. The use of profiles is familiar to molecular biologists from such tasks as prediction of gene structure by homology, delineation of protein families, and fold recognition. The same intuition applies to iterative search of vector spaces for similarities between gene vectors. Because query vectors are converted into probabilistic models that can be iteratively updated, the resulting sensitivity of the method is higher than in simple similarity searches.

Some of the ideas that were used in psi-square algorithm have been discussed before. Most notably, Zhou and co-authors [51] have introduced the shortest path concept, which seeks to find the series of closest neighbors in genome-wide data in an iterative fashion. In contrast to their approach, psi-square does not rely on pre-computed network, but uses a query to interrogate unordered vector space and to produce a probabilistic model of the query. Our approach also estimates the significance of observed similarities from the background data, similar to what is done in sequence database searches.

The performance of psi-square depends on the choice of distance/similarity measure and several search parameters, most notably *K, r*, and *s*. The optimal choice of distance/similarity measure in genome-wide datasets is an important problem, which we examine elsewhere [52]. The need to choose the value of *K*, and the very idea of discretization of numerical data, may feel counterintuitive, as discretization is usually thought to lead to the loss of information. Recently, however, has been shown that glioma tumor types can be perfectly separated using binarily receded expression vectors [53], confirming that this data transformation preserved high information content of expression vectors. Several efficient methods to extract information from the data, e.g., Boolean analysis, work only in binary domain, and successful application of this technique to binary receded expression vectors was recently described [54].

In our hands, the naïve, unsupervised equal-width interval binning discretization proved superior to other schemes of data transformation (data not shown). Novel way to transform data for psi-square search may be developed in the future.

The study of the *r *and *s *parameter space associated with different types of genome-wide datasets is another venue for future research. We expect that the optimal values of *r *and *s *will be highly dependent on the data.

Our initial attempts to correct the potential bias of zero counts by including pseudocounts degraded the performance of the program in Problem 1. We think that this may, in part, be related to the low dimensionality of each column vector in DSSM. The effect of various smoothing schemes on psi-square sensitivity and selectivity, nevertheless, deserves further investigation.

In conclusion, we proposed the similarity search program, psi-square, which is applicable to probabilistic matching of any patterns represented in the vector form.

## Materials and methods

### Psi-square algorithm

Psi-square algorithm proceeds in five steps.

1. Initialize the program with a vector or a group of related vectors (initial value of target vector set). The algorithm proceeds further if there are at least three vectors similar to the query at the given similarity threshold (*r *value).

2. Construct the dimension-specific scoring matrix (DSSM) of the form *s*_*kj *_= log(*f*^*T*^_*kj*_*/f*^*B*^_*kj*_), where *k *varies over the number of possible values of vectors (or transformed vectors, see below) and *j *varies over the set of vector coordinates.

3. Use the DSSM as a query at the next iteration of the search. Score similarity between DSSM and each database vector as follows: *S*(vector) = ∑ *s*_*kj*_, where *s*_*kj *_is the score of value *k *at vector coordinate *j*. Vectors with higher similarities to DSSM get higher scores. Construct the empirical distribution of these scores. Record vectors with scores from a given percentile (e.g., 99^th^) of the total score distribution as new high-scoring matches.

4. Add these vectors to the target vector set; update the DSSM.

5. Repeat step 3. The process terminates when we cannot find new matches at step 3.

The vectors' coordinates can be either discrete or continuous. Discrete coordinates often have only two states, e.g., "turned on-turned off" or "present-absent", but they may be multistate. The present algorithm assumes a finite number of states, which can be achieved by discretizing continuous variables. Discretization simplifies the data representation, and some machine-learning algorithms have been shown to perform better with discrete-valued attributes, even though they can also handle continuous attributes [55,56]. We used the simples feature disretization strategy, i.e., unsupervised equal-width interval binning, dividing the range of observed values for a variables into *K *equal sized bins [57]. *K *can be either dictated by an *ad hoc *scientific hypothesis, or computed on the fly, as explained below.

For every vector in the database, the range of its values, *E*_*max *_*and E*_*min *_is calculated with step δ = *E*_*max*_*-E*_*min*_*/K*. Each vector is transformed to receive a set of discretized coordinates, where its *i*^th ^value is replaced by the attribute (). The number of intervals depends on the data set. For example, in sequence similarity analysis, the number of initial states for nucleic acids may be naturally set for five – four nucleotides and the gap. For coded binary character states, such as presence/absence, *K *is 2. The value of *K *is estimated from the initial target vector set, by minimizing the distortion between the discretized initial target vector set over 1 <*k *<*K*_*l*_, where 2 <*K*_*l *_<*N*, *m *is the number of patterns in the initial target vector set, *l *is the overall number of different intervals we try, and is the transformed *i*^th ^vector.



For every query *K *is estimated at the initial iteration. In the sequel as well as in the psi-square software, we use the Euclidean distance *d*_*E *_between (,) as our distance function.

There are two more parameters that have to be specified, the similarity threshold for the inclusion in the target vector set, *r*, in step 1, and the percentile of the score distribution that is used as the inclusion cutoff *s*, in step 3. The optimal values of *r *and *s *depend on the sample size and signal-to-noise ratio in the data, and their selection is similar to the decisions commonly made in sequence database searches. For convenience, psi-square software allows one to construct the distribution of similarity measures and choose the threshold empirically. In all examples we were using conservative 99.9^th ^percentile of score distribution as the threshold for *s*, in order to avoid the explosion of false positives.

### Phyletic vectors

Gene presences and absences are summarized in the COG database . There were 4873 COGs from 66 complete genomes of unicellular organisms in the COG database, as of September 21, 2004 [22]. 284 fungi-specific COGs were not considered in this study. Each *i*th COG (*i = *1,...,4589) is a phyletic vector, where the *j*th coordinate (*j *= 1,...,63) is set at 1 if it is represented in the *j*th genome and 0 if it is not (we ignore some details, such as the presence of in-paralogs in some COGs – see [22] for discussion). In this case *K *is set at 2, corresponding to two possible values of binary coordinates, 0 and 1. We were using 98^th ^(*r *= 0.6) and 99^th ^(*r *= 0.7) percentiles of correlation coefficient distribution as the thresholds for similarity measure for psi-square with simple and combined queries, respectively.

### Protein-protein interaction vectors

We used the tandem-affinity purification (TAP) data set from Gavin et al. [45] and removed purifications that only retrieved the bait itself. This retains 455 purifications, containing 1361 proteins. The K parameter is naturally set at 2. In this example, we were using 99.5^th ^(*r = 0.6*) percentile of correlation coefficient distribution as the threshold for similarity measure.

### Gene expression vectors

Gene expression data for the asexual intraerythrocytic developmental cycle (IDC) of the malaria parasite *P.falciparum *are from Bozdech et al. [40] (Quality Control data set, 5081 vectors with 46 coordinates). Missing data and outliers (coordinates deviating more than 3 s.d. from the mean value for a given vector) were replaced by the mean; this is called "the IDC set" in the sequel. The parameters for this data were chosen iteratively, in order to maximize the number of new matches and minimize the average number of matches.

### Specificity and sensitivity estimates

When the training sample (list of proteins with desired properties) is available, we compare the sensitivity and specificity of psi-square with the performance of the approaches used in the literature for each analysis. Specificity is computed as TP/(TP+FP) and sensitivity as TP/(TP+FN), where TP denotes true positives (genes/proteins included in the training sample); FP denotes false positives (genes/proteins not included in the training sample), and FN denotes false negatives (genes/proteins included in the training sample but not found by the approach).

### Software availability

Psi-square code and formatting utilities are at 

## Reviewers' comments

### Reviewer's report 1

*I*. *King Jordan, National Institutes of Health, Bethesda, MD, USA*

Glazko *et al*. present a new program – *psi-square *– for analyzing 'gene vectors'. The gene vectors considered by *psi-square *can be made up of quantitative measures of a number of gene attributes, such as expression levels, phyletic profiles and fitness effects, which are generated by high-throughput functional and/or comparative genomics studies. Given such a set of measures for any query gene, one may wish to identify sets of genes with similar values – *i.e*. neighbors of the query vector in high-dimensional space. Identification of such neighbors can reveal functional and evolutionary links between genes or membership in biochemical pathways. Thus, the manuscript tackles a critical analytical problem of the post-genomics era.

The program *psi-square *derives its name from the widely employed sequence similarity program PSI-BLAST. Like PSI-BLAST, *psi-square *employs an iterative search strategy: the query gene defines a numerical pattern (vector) of interest, a group of highly similar vectors is then identified via a database search, a probabilistic model of the resulting group is derived and the database search is then repeated to identify other similar vectors. The process is repeated until it converges. The authors applied *the psi-square *approach to three specific genomic problems – phyletic distributions of orthologs, gene expression vectors and protein interaction profiles – and demonstrate that it performs with greater sensitivity than previously employed methods. The specificity of the method, however, is not always superior as may be expected for the kind of iterative strategy that it uses. Indeed, a critical component of iterative sequence analysis strategies is user specified decisions as to what sequences should and should not be included in the set used to build a profile. The inclusion of such intelligent user input into the *psi-square *algorithm should also allow for improved specificity.

**Author response: ***We agree that the option for user specified filtering of new matches to make more specific profiles for further iterations may significantly increase the specificity of psi-square. We may consider this option in the future software development, and would like to also note that the decisions of what to include in a DSSM are analogous to the construction of PSSM in sequence analysis, where often this has to be done outside of the iterative search (though web interface of PSI-BLAST provides some control)*.

*Psi-square *seems like a very promising method for the meaningful comparison of gene attribute vectors. In some sense though, this work seems a bit preliminary. There are many issues that can affect the performance of the algorithm and few of these have been ironed out just yet. For instance, the performance of the program depends critically on the distance measure used to compare vectors. The authors have dealt with this issue elsewhere, but the choice of the appropriate distance measure could be far from obvious for the user. There are also several other search parameters that need to be chosen carefully, and the optimal values of these parameters for different kinds of data are not known.

**Author response: ***Any problem involving computation of distances tends to be sensitive to the choice of distance measure. We provide a healthy spectrum of different distance measures that can be chosen by the user, not necessarily blindly. For more discussion of the choice of distance measure, one may consult, for example, Glazko et al, 2005 (Pubmed 16306389)*.

*Psi-square *employs log-odds ratios, analogous to sequence comparisons, of probabilities of vector scores in the target set (query) over the background set (database). Since these probabilities are estimated as frequencies, this approach would seem to hinge upon the size of the target database, in terms of the robustness of the frequency estimates. This dependence has not been evaluated here.

**Author response: ***The size of biologi cal data sets we had in mind when designed psi-square (gene expression vectors, phyletic vectors, etc.) is on the order of tens of thousands, therefore the frequencies estimates should be robust. We agree, however, that further examination of different datasets is worthy of investigation*.

In addition, the examples in this manuscript deal solely with gene vectors based on single attributes. It would be most interesting to see how the method performs on gene vectors that combine quantitative measures of different gene attributes, such as gene expression combined with phyletic profile data. The authors are entirely transparent with respect to these kinds of issues though and do address them in the manuscript. So this relatively mild critique should not be taken as a refutation of the utility or relevance *of psi-square*. Rather, it is simply a caveat that much needs to be done to get *psi-square *to the point where it can maximize its value to working biologists.

Perhaps more relevant to the biological community is the fact that *psi-square *is not yet ready for prime time in terms of being a widely used application. To their credit, the authors make *psi-square *freely available on their website. However, the installation and implementation of the program are far from simple and probably beyond the ability of the many of the bench scientists who would benefit most from its use. Unfortunately, the command line interface alone will rule out the use of this program for many working biologists. It would be nice to see *psi-square *implemented as a web server, for instance, where users could select settings and parameter values from drop-down menus. The program would also benefit from a windows interface. I suspect that many of these issues will be worked out as and the importance of systematically revealing functional connections between genes becomes increasingly apparent and the methods to achieve this are developed accordingly.

**Author response: ***We will gauge the users' response to define further software development. We count on some level of initial acceptance by those familiar with the BLAST commandline interface*.

### Reviewer's report 2

Mikhail Gelfand, Institute of Information Transfer Problems, Moscow, Russia

The paper is very interesting and may be published "as is", aside of a minor editorial comment. The authors use the modern way of putting "Methods" at the end, which creates difficulties for a paper whose main substance is a new method. One consequence is that parameters such as S and R are used (without explanation) before they are defined. I suggest either adding such explanation (leading to some repetition) or re-organizing the paper so that "Methods" include only very formal description of databases used etc., whereas the description of the algorithm is placed under "Results". Personally, I think the latter is more sensible, but it clearly is a matter of taste. Formally, I suggest publishing the paper with my editorial endorsement, but without a comment which is clearly technical.

**Author response: ***We added the definitions for r, s parameters on the p. 6 of the manuscript*.

### Reviewer's report 3

Nicolas Galtier, CNRS-Université Montpellier II, Montpellier, France

This manuscript introduces a new method for extracting similarity information from genome-wide multidimensional data sets – typically the expression pattern of many genes. The algorithm is related to psi-blast in relying on an iterative search using an evolving query profile. The goal is to extract from a large data base those vectors similar to some query vector.

I liked reading this manuscript, and I think is is a valuable contribution to bioinformatics. The addressed problem is presumably becoming more and more frequent in the practice of biologists. The method appears globally sound to me, although some details must be clarified (see below). The three examples given are relatively convincing that the method will be useful.

The only reserve I have is that the method requires arbitrarily defined parameters, namely r, s and K. This is not a problem in general (users will appreciate to be able to play with s depending on the specific task they face), but it makes the comparison with other programs a bit unclear.

Now questions about the method:

- I did not understand what r means. The second sentence of step 1 in Material and method is mysterious to me. Some similarity appears to be calculated between vectors before computing the DSSM. I did not follow this part of the algorithm. Isn't the target vector set equivalent to the query, and therefore just user-defined?

**Author response: ***We use r as the initial threshold to select the target set of vectors with the highest similarity to the query vector. It is used as a threshold only at this first iteration, and the inclusion of other vectors into DSSM in further iterations is governed by another parameter, s. We resorted for this two-parameter scheme for now, even though it is possible that analysis of the parameter space will allow to generalize to just one parameter in the future. An alternative, implemented in PSI-BLAST, is to allow the user to redefine a similar parameter (-h) at each iteration*.

- The definition of the similarity between the query and any vector -

S(vector) = sum{s_{kj}} – is unclear. I suggest to call (transformed) vector V, to explain that k is equal to V_j, and that the summation is over j. Or you could avoid using k and just write S(V) = sum_jj}} – well, if this is how you actually define similarity scores.

**Author response: ***In some cases (for binary vectors), transformed and untransformed vectors are the same, so, for the sake of simplicity, we did not introduce the special notation for transformed vectors (except in the very end of the method section, where we demonstrate how to choose the number of categories)*.

- I am surprised that threshold s is defined as a percentile. It seems to me that the program will return exactly M.(1-s) vectors whatever the distribution of similarity scores, i.e., even if the number of vectors truly similar to the query is much less (or more) than that. Is this true? Does it make any sense to put the threshold on the similarity score (perhaps divided by N)?

**Author response: ***After the initial step the similarity to the query profile is defined only through scores, which have some distribution. All database vectors are scored, but we only those with the highest scores are of further interest. That is we implicitly define via this threshold how many database vectors will be returned as an output and included in the profile on the next iterations*.

- It would be useful to give the complexity of each step of the algorithm.

**Author response: ***The worst case scenario complexity for the entire algorithm is O(n*^2^*)*.

### Reviewer's report 4

Sarah Teichmann, MRC Laboratory of Molecular Biology, Cambridge, United Kingdom

This manuscript by Glazko et al. on a method for identifying profile similarities for genome-wide data sets represents an interesting advance from pair-wise to multiple profile comparisons. The iterative profile comparison program psi-square can be applied to any query vector on a database of profiles. The types of data presented for use of the program are expression data, phyletic vectors and affinity purifications of protein complexes. The utility of the program for each of these data sets is illustrated with a particular example, such as detection of bacterial flagellar components.

While these illustrative examples are compelling, a more large-scale benchmark of the program would be desirable. Examples of previous benchmarking efforts are consistency of COG functional categories in Bowers PM *et al*. (2004). Huynen et al. (2000, *Genome Res*) benchmarked different non-homology methods for function predictions using known functional relationships in the minimal *M. genitalium *genome, including physical interactions, metabolic pathways, regulatory relationships *etc*. Something along these lines would be more satisfactory than simply extracting individual model examples as benchmarks.

My second query is about the different parameters that would be appropriate for different data types and different data sets. The method is clearly general, but at the same time there are likely to be very different optimal parameters depending on the situation. Some guidelines for these through benchmarks would be useful. In the same vein, it is mentioned that integration of different data sets would be possible with psi-square. However, there is no further discussion of the implications and biological meaning of this and how this would be carried out in practise.

**Author response: ***We agree with the reviewer that the examples presented are case studies. This is because the main point of our work is not in benchmarking different genomic context methods (as in Huynen et al., 2000), but in introduction and initial justification of general approach to pattern matching in binary vector space. Our examples serve to demonstrate that psi-square works with any data space that takes a vector form, which makes it applicable to a broad range of problems in genomics – the problems that tended to be solved by ad hoc or domain-specific (and sometimes less successful) methods before. We agree that large-scale benchmarking would be desirable and necessary in the future, also given that the psi-square parameter space and choice of distance measures are already extensive. As for combining several attributes (as another reviewer has also mentioned), it can be done naively even now, by simply concatenating different vectors associated with the same set of genes; but the optimal way of integrating information from diverse gene vectors deserves a separate study*.

Despite the two criticisms mentioned above, this program is a promising advance over simple pairwise comparison of vectors of functional or phyletic data and clearly has widespread application. Making the method available as a downloadable program or a webserver would undoubtedly prove very popular.

## Supplementary Material

Additional data file 1 Figure, showing proteins, associated with flagella phenotype, identified by simple psi-square and PP algorithms. Diagram: 45 COGs identified by psi-square when COG1298 was used as a query; 37 COGs related to flagella biogenesis and function; 46 COGs identified by PP algorithm. Click here for file

Additional data file 2 Figure, showing proteins associated with flagella phenotype, identified by psi-square with combined query and TTG algorithms. Diagram: 73 COGs identified by psi-square; two other sets are the same as for Figure 2 in the main text. Click here for file

Additional data file 3 Tables 1, 2, 3, 4. Click here for file

Additional data file 4 Figure, showing a) Factors likely regulating Poly(A)-tail synthesis and maturation, found by psi-square using Ptal as a query. Graph vertices are connected only if the corresponding proteins were found in the same purification. b) The intersection of three protein sets: 18 proteins found by psi-square when Ptal was used as a query; 20 proteins identified as factors regulating Poly(A)-tail synthesis and maturation by Gavin et al. (2002); 33 proteins found by psi-square with purification-made query. Click here for file

Additional data file 5 Figure showing factors likely regulating Poly(A)-tail synthesis and maturation, found by psi-square using purification as a query. Click here for file
