# Supplementary material for: Similarity searches in genome-wide numerical data sets
Source: Biol Direct. 2006 May 30;1:13. doi: 10.1186/1745-6150-1-13 (PMC1489924; doi:10.1186/1745-6150-1-13)

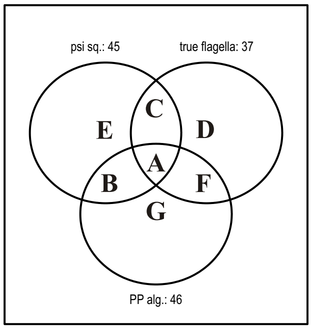

Figure. Proteins, associated with flagella phenotype, identified by simple psi-square and PP algorithms. Diagram: 45 COGs identified by psi-square when COG1298 was used as a query; 37 COGs related to flagella biogenesis and function; 46 COGs identified by PP algorithm

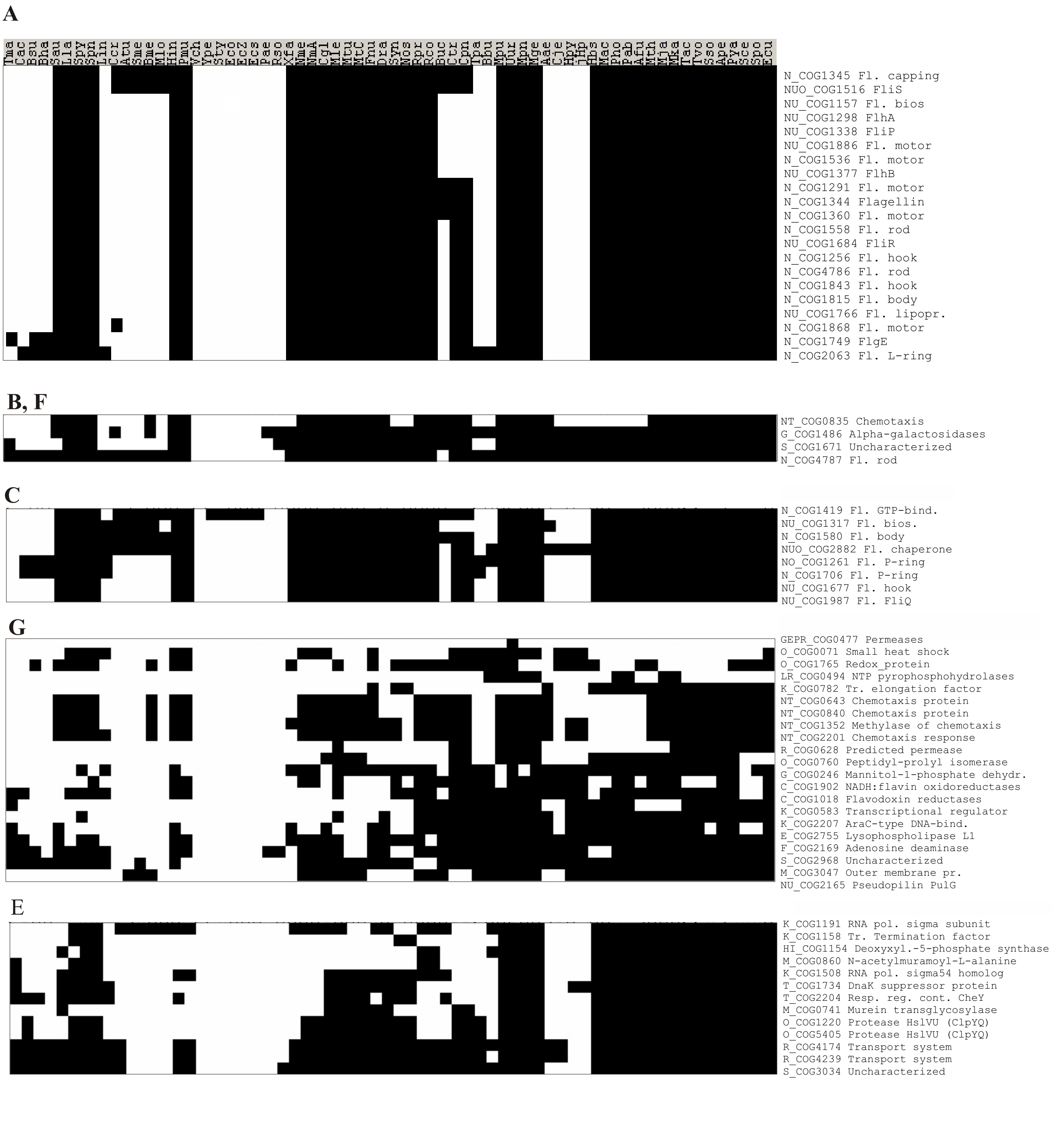

Supplement: Additional data file 1 — Figure, showing proteins, associated with flagella phenotype, identified by simple psi-square and PP algorithms. Diagram: 45 COGs identified by psi-square when COG1298 was used as a query; 37 COGs related to flagella biogenesis and function; 46 COGs identified by PP algorithm. [file 1745-6150-1-13-S1.pdf]
