# Supplementary material for: Similarity searches in genome-wide numerical data sets
Source: Biol Direct. 2006 May 30;1:13. doi: 10.1186/1745-6150-1-13 (PMC1489924; doi:10.1186/1745-6150-1-13)

Figure. Proteins associated with flagella phenotype, identified by psi-square with combined query and TTG algorithms. Diagram: 73 COGs identified by psi-square; two other sets are the same as for Figure 2 in the main text.

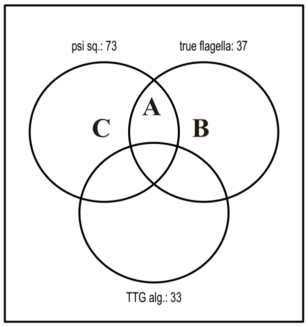

A

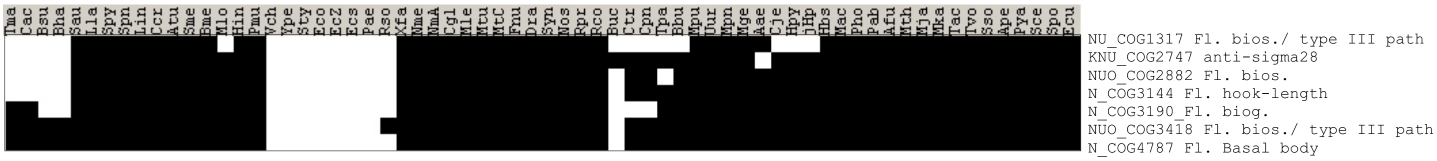

B

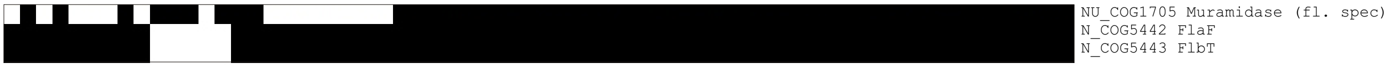

C

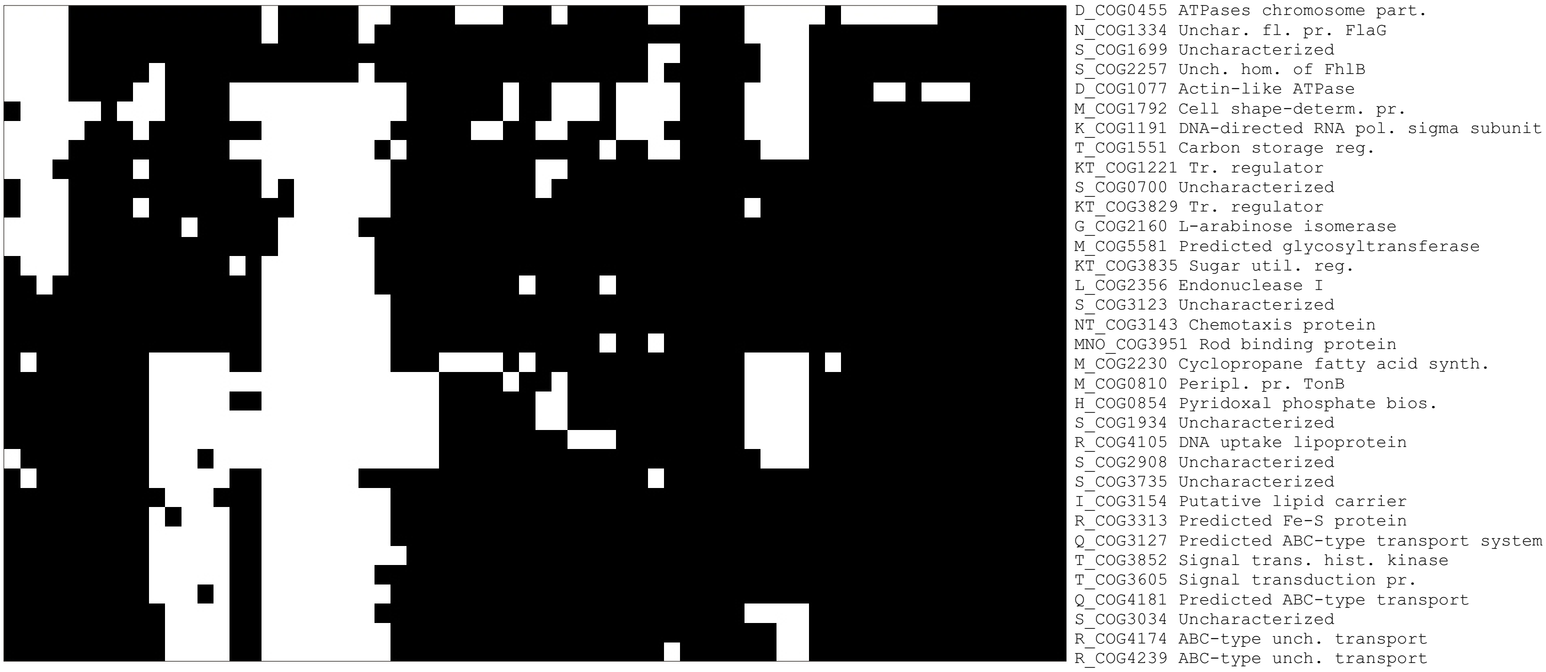

Supplement: Additional data file 2 — Figure, showing proteins associated with flagella phenotype, identified by psi-square with combined query and TTG algorithms. Diagram: 73 COGs identified by psi-square; two other sets are the same as for Figure 2 in the main text. [file 1745-6150-1-13-S2.pdf]
