# Supplementary material for: Similarity searches in genome-wide numerical data sets
Source: Biol Direct. 2006 May 30;1:13. doi: 10.1186/1745-6150-1-13 (PMC1489924; doi:10.1186/1745-6150-1-13)
Supplement: Additional data file 3 — Tables 1, 2, 3, 4. [file 1745-6150-1-13-S3.pdf]

**Table 1. Unreported connections to flagellar phenotype based on contextual information from STRING database.**

| COG number | STRING prediction |
|---|---|
| COG1699: Uncharacterized protein conserved in bacteria | Prediction is based on conserved neighborhood: COG1344, flagellin and related hook-associated protein |
| COG2257: Uncharacterized homolog of the cytoplasmic domain of flagellar protein FhlB | Prediction is based on coexpression with COG1344, flagellin and related hook-associated proteins |
| COG3034: Uncharacterized protein conserved in bacteria | Prediction is based on coexpression with COG2257, uncharacterized homolog of the cytoplasmic domain of flagellar protein FhlB |

**Table 2. The average number of iterations, unique matches (detected by psi-square alone), and matches found by both psi-square and 5%-ED method.**

| CORR./K | IT | MATCHES | SHARED | PSI-SQUARE ONLY | 5%ED ONLY |
|---|---|---|---|---|---|
| cor07_step05 | 1.1 | 508.3 | 418 | 605 | 1 |
| cor07_step10 | 2.1 | 513.6 | 418 | 605 | 1 |
| cor07_step15 | 1.7 | 509.4 | 418 | 605 | 1 |
| cor07_step20 | 1.3 | 508.4 | 418 | 605 | 1 |
| cor07_step25 | 1.1 | 508.3 | 418 | 605 | 1 |
| cor07_step30 | 1.1 | 508.3 | 418 | 605 | 1 |
| cor07_step35 | 1 | 508.1 | 418 | 605 | 1 |
| cor07_step40 | 1 | 508.1 | 418 | 605 | 1 |
| cor08_step05 | 1.7 | 339.7 | 418 | 356 | 1 |
| cor08_step10 | 3.7 | 352.7 | 418 | 358 | 1 |
| cor08_step15 | 2.1 | 339.1 | 418 | 356 | 1 |
| cor08_step20 | 2.4 | 338.1 | 418 | 356 | 1 |
| cor08_step25 | 1.3 | 334.9 | 418 | 356 | 1 |
| cor08_step30 | 1.6 | 335.9 | 418 | 356 | 1 |
| cor08_step35 | 1.4 | 336 | 418 | 356 | 1 |
| cor08_step40 | 1.3 | 335 | 418 | 356 | 1 |
| cor09_step05 | 3.3 | 193.1 | 408 | 125 | 11 |
| cor09_step10 | 3.3 | 188.1 | 409 | 169 | 10 |
| **cor09_step15** | **4** | **196.4** | **409** | **187** | **10** |
| cor09_step20 | 3.1 | 189.3 | 410 | 160 | 9 |
| cor09_step25 | 2 | 179.3 | 408 | 152 | 11 |
| cor09_step30 | 2.4 | 183 | 407 | 150 | 12 |
| cor09_step35 | 1.9 | 180.6 | 407 | 164 | 12 |
| cor09_step40 | 2 | 177.4 | 407 | 151 | 12 |

**Table 3. Properties of proteins in two HPs set. Proteins in $HP_{s1}$ were found only by psi-square; and proteins in $HP_{s2}$ were found by both, psi-square and 5%ED approaches.**

| SET | Protein length | TM regions | %Loop regions | %Helical regions |
|---|---|---|---|---|
| $HP_{s1}$ | 888.39±8.33 | 1.17±0.02 | 91.08±0.13 | 8.92±0.13 |
| $HP_{s2}$ | 929.08±6.85 | 1.28±0.02 | 94.09±0.07 | 5.85±0.07 |

**Table 4. Predicted molecular functions for the two groups of proteins.**

| HPs1 set: proteins found only by psi-square | | | HPs12 set: proteins found by both: the psi-square and 5%ED methods | | |
|---|---|---|---|---|---|
| **ID in PlasmoDB** | **Ortholog in Plasmodium yoelii (1: presence, 0: absence)** | **Putative function (based on PSI-BLAST score)** | **ID in PlasmoDB** | **Ortholog in Plasmodium yoelii (1: presence, 0: absence)** | **Putative function (based on PSI-BLAST score)** |
| MAL13P1.102 | 1 | | MAL13P1.109 | 1 | Protein kinase domain |
| MAL13P1.152 | 1 | | MAL13P1.114 | 1 | Protein kinase domain |
| MAL13P1.158 | 1 | | MAL13P1.130 | 1 | |
| MAL13P1.268 | 0 | Distantly related to portion of CLAG domain | MAL13P1.188 | 1 | |
| MAL6P1.100 | 1 | | MAL13P1.228 | 1 | |
| MAL6P1.121 | 0 | Tetrahydrofolate dehydrogenase/cyclohydrolase | MAL13P1.278 | 1 | Protein kinase domain |
| MAL6P1.127 | 1 | Enzyme with alpha/beta hydrolase fold | MAL13P1.308 | 1 | Armadillo/beta-catenin repeats |
| MAL6P1.174 | 1 | | MAL13P1.328 | 1 | |
| MAL6P1.200 | 1 | | MAL13P1.94 | 1 | |
| MAL6P1.63 | 0 | | MAL6P1.145 | 1 | CDP-alcohol phosphatidyltransferase (probably ethanolamine or choline-specific) family |
| MAL7P1.13 | 0 | | MAL6P1.147 | 1 | HECT-domain ubiquitination enzyme |
| MAL7P1.167 | 0 | | MAL6P1.178 | 1 | |
| MAL7P1.34 | 0 | | MAL6P1.216 | 1 | TPR domain |
| MAL8P1.160 | 1 | | MAL6P1.247 | 1 | |
| MAL8P1.4 | 1 | | MAL6P1.286 | 1 | Myosin-like coiled coil |
| MAL8P1.88 | 0 | PFEMP-like protein? | MAL6P1.289 | 1 | |
| PF07_0014 | 0 | | MAL6P1.299 | 1 | membrane protein PF12 |
| PF07_0107 | 0 | | MAL7P1.119 | 0 | |
| PF08_0082 | 1 | | MAL7P1.125 | 1 | |
| PF08_0083 | 0 | | MAL7P1.84 | 1 | |
| PF08_0091 | 1 | | MAL8P1.106 | 1 | |
| PF08_0115 | 1 | DnaJ domain | MAL8P1.109 | 1 | Protein phosphatase 2C |
| PF08_0118 | 1 | | MAL8P1.12 | 0 | |
| PF10_0023 | 1 | | MAL8P1.13 | 1 | |

| | | | | | |
|---|---|---|---|---|---|
| PF10_0098 | 0 | | MAL8P1.150 | 1 | Adenylate or Guanylate cyclase |
| PF10_0100 | 1 | | MAL8P1.73 | 1 | |
| PF10_0157 | 0 | | PF07_0072 | 1 | Protein kinase with calmodulin domain |
| PF10_0163 | 0 | | PF07_0127 | 1 | |
| PF10_0170 | 1 | | PF08_0129 | 1 | Protein phosphatase 2B-like, calcineurin-like phosphoesterase |
| PF10_0189 | 1 | Cys finger | PF10_0034 | 1 | |
| PF10_0223 | 1 | Cys finger | PF10_0037 | 1 | |
| PF10_0231 | 1 | | PF10_0094 | 1 | Tubulin-tyrosine ligase family |
| PF10_0296 | 0 | | PF10_0119 | 1 | |
| PF11_0151 | 1 | TBC1 domain, Rab GTPase activator | PF10_0135 | 0 | |
| PF11_0300 | 1 | | PF10_0138 | 1 | |
| PF11_0304 | 1 | | PF10_0156 | 1 | |
| PF11_0398 | 0 | | PF10_0193 | 1 | Microtubule associated protein 1A/1B, light chain, aka autophagy 8i protein |
| PF11_0402 | 1 | RRM RNA recognition motif | PF10_0220 | 1 | Phospholipid scramblase |
| PF11_0441 | 1 | Eenzyme with alpha/beta hydrolase fold | PF10_0281 | 1 | NOA36-like repeats, also in microneme protein 4 |
| PF11_0513 | 1 | DnaJ domain | PF10_0295 | 1 | |
| PF11_0528 | 1 | | PF10_0306 | 1 | Membrane Occupation and Recognition Nexus (MORN) repeat |
| PF13_0012 | 1 | ETRAMP | PF10_0308 | 1 | OTU-like cysteine protease |
| PF13_0031 | 1 | | PF10_0345 | 1 | |
| PF13_0072 | 1 | | PF10_0348 | 1 | PFEMP-1 family |
| PF13_0079 | 1 | | PF10_0352 | 1 | |
| PF13_0159 | 1 | Nucleotidyl transferase, possibly nicotinate-nucleotide adenylyltransferasePF13_0162 | PF10_0367 | 0 | |
| PF13_0162 | 1 | | PF10_0368 | 1 | Dynamin GTPase effector domain |
| PF14_0013 | 1 | DnaJ domain | PF11_0060 | 1 | Ca/calmodulin-dependent protein kinase domain |
| PF14_0016 | 1 | ETRAMP | PF11_0094 | 1 | |
| PF14_0044 | 1 | | PF11_0168 | 1 | Lipase/serine esterase |
| PF14_0045 | 1 | | PF11_0180 | 1 | |
| PF14_0108 | 1 | | PF11_0193 | 0 | |
| PF14_0250 | 1 | Lipase class 3 | PF11_0194 | 1 | |
| PF14_0291 | 0 | | PF11_0204 | 0 | |
| PF14_0344 | 0 | | PF11_0277 | 1 | |
| PF14_0472 | 0 | | PF11_0278 | 1 | |
| PF14_0525 | 1 | Dual specificity protein phosphatase | PF11_0287 | 1 | CRAL/TRIO lipid-binding domain |

| | | | | | |
|---|---|---|---|---|---|
| | | (S/T and Y) | | | |
| PF14_0574 | 1 | Zn finger, FYVE-type | PF11_0344 | 1 | pfam02430, AMA-1, Apical membrane antigen 1 |
| PF14_0594 | 0 | | PF11_0373 | 1 | PFEMP |
| PF14_0712 | 1 | | PF11_0379 | 1 | |
| PF14_0733 | 1 | | PF11_0383 | 1 | |
| PF14_0734 | 1 | Protein kinase domain | PF11_0415 | 1 | |
| PF14_0760 | 0 | | PF11_0417 | 0 | |
| PFA0135w | 1 | Tryptophan-rich secreted blood-stage antigen | PF11_0464 | 1 | pfam00069, pkinase, Protein kinase domain |
| PFA0215w | 0 | | PF11_0474 | 0 | |
| PFA0635c | 0 | | PF13_0115 | 1 | |
| PFB0105c | 1 | | PF13_0173 | 1 | |
| PFB0106c | 0 | | PF13_0197 | 1 | merozoite surface protein 7 precursor |
| PFB0120w | 1 | ETRAMP | PF13_0255 | 1 | |
| PFB0145c | 1 | | PF13_0256 | 1 | |
| PFB0190c | 1 | TPR/SEL-1 repeat | PF13_0314 | 1 | PFEMP |
| PFB0194w | 1 | | PF13_0326 | 0 | pfam00241, cofilin_ADF, Cofilin/tropomyosin-type actin-binding protein |
| PFB0485c | 0 | | PF14_0021 | 1 | |
| PFB0900c | 1 | | PF14_0102 | 1 | Pr86 rhoptry precursor protein |
| PFB0910w | 1 | Merozoite Surface Antigen 2 (MSA-2) family | PF14_0135 | 1 | |
| PFC0090w | 1 | | PF14_0149 | 1 | |
| PFC0695w | 1 | | PF14_0172 | 1 | |
| PFC0930c | 1 | | PF14_0173 | 1 | pfam00027, cNMP_binding, Cyclic nucleotide-binding domain |
| PFD0495c | 1 | | PF14_0196 | 1 | TPR Domain |
| PFD0705c | 1 | | PF14_0224 | 1 | PP1-like serine/threonine phosphatase, calcineurin-like |
| PFD0985w | 1 | PR repeat | PF14_0293 | 0 | |
| PFD1135c | 0 | Solute carrier family 5 (choline transporter) | PF14_0333 | 1 | |
| PFD1165w | 1 | Protein kinase domain | PF14_0334 | 1 | Glutamate synthase, FMN-binding domain |
| PFE0360c | 1 | | PF14_0346 | 1 | cyclic nucleotide-dependent protein kinase |
| PFE1255w | 1 | SMC-RecF/N family ATPases (chromosome segregation) | PF14_0388 | 1 | |
| PFE1505w | 0 | | PF14_0495 | 1 | |
| PFI0120c | 1 | Protein kinase domain | PF14_0572 | 1 | |

| | | | | | |
|---|---|---|---|---|---|
| PFI0845w | 1 | | PF14_0578 | 0 | |
| PFI0850w | 0 | Phosphatidylserine decarboxylase | PF14_0586 | 1 | MORN domain |
| PFI1200w | 0 | | PF14_0607 | 1 | |
| PFI1520w | 0 | | PF14_0652 | 1 | D13 Zn-finger protein |
| PFI1720w | 0 | | PF14_0660 | 1 | Ser/Thr phosphatase, calcineurin-like |
| PFL0065w | 0 | | PF14_0694 | 0 | thioredoxin, protein disulphide isomerase |
| PFL0275w | 1 | Zinc finger, C3HC4 type (RING finger) | PF14_0732 | 1 | |
| PFL0800c | 1 | | PFB0150c | 1 | pfam00069, protein kinase domain |
| PFL0895c | 1 | Dynamin family GTPase (role in endocytosis) | PFB0305c | 1 | merozoite surface protein MSP5 |
| PFL1055c | 0 | | PFB0475c | 1 | |
| PFL1945c | 1 | ETRAMP | PFB0570w | 1 | annotated as "altered tromposprondin domain", in fact not so similar to it |
| PFL2530w | 1 | alpha/beta hydrolase fold, probably lysophospholipase | PFB0680w | 1 | |
| PFL2565w | 1 | | PFB0815w | 1 | protein kinase with EF-hand |
| MAL13P1.329 | 1 | | PFB0935w | 1 | CLAG, Cytoadherence-linked asexual protein |
| MAL6P1.300 | 1 | PP-loop superfamily ATPase-pyrophosphatase | PFC0120w | 1 | CLAG, Cytoadherence-linked asexual protein |
| PF07_0011 | 1 | Modified HMA (heavy metal chaperone) domain | PFC0560c | 1 | |
| PFC0150w | 1 | Lipin, mutations cause fatty acid depletion in mice | PFC0820w | 1 | |
| PFD0715c | 1 | | PFC0945w | 1 | pfam00069, pkinase, Protein kinase domain |
| PFE0200c | 1 | Zinc finger (AN1-like) family | PFD0100c | 1 | |
| PFE0340c | 1 | Rhomboid-family intramembrane protease | PFD0105c | 1 | |
| PFE0070w | 1 | | PFD0195c | 1 | |
| | | | PFD0230c | 1 | Papain family cysteine protease |
| | | | PFD0295c | 1 | pfam00084, sushi, Sushi domain (SCR repeat) |
| | | | PFD0380c | 1 | |
| | | | PFD0385w | 1 | |
| | | | PFD0390c | 0 | AAA family ATPase |
| | | | PFD0955w | 0 | |
| | | | PFD1100c | 1 | |
| | | | PFE0045c | 1 | pfam00069, pkinase, Protein kinase domain |

| | | | | | |
|---|---|---|---|---|---|
| | | | PFE0075c | 1 | rhoptry-associated protein |
| | | | PFE0080c | 1 | rhoptry-associated protein |
| | | | PFE0175c | 1 | Myosin head motor domain ATPase (NOT non-specific coiled coil domain) |
| | | | PFE0365c | 1 | |
| | | | PFE0770w | 1 | |
| | | | PFE1105c | 1 | |
| | | | PFE1120w | 1 | |
| | | | PFE1130w | 1 | DUF803 family, maybe a transporter most similar to nucleotide-sugar transporters |
| | | | PFE1260c | 1 | |
| | | | PFE1410c | 1 | |
| | | | PFE1415w | 0 | DHHC/NEW1 zinc finger domain, including palmitoyl transferases |
| | | | PFI0175w | 1 | |
| | | | PFI0265c | 1 | rhoptry complex protein |
| | | | PFI0360c | 1 | |
| | | | PFI0410c | 1 | |
| | | | PFI0540w | 1 | |
| | | | PFI0690c | 1 | |
| | | | PFI0785c | 1 | Transporter, major facilitator superfamily |
| | | | PFI1005w | 1 | ADP-ribosylation factor family |
| | | | PFI1015w | 1 | CRAL/TRIO lipid-binding domain |
| | | | PFI1445w | 1 | Rhoptry complex protein |
| | | | PFI1475w | 0 | Merozoite surface protein MSP-1 |
| | | | PFI1550c | 1 | |
| | | | PFI1685w | 1 | Protein kinase C family |
| | | | PFL0300c | 0 | Ser/Thr protein phosphatase, calcineurin-like |
| | | | PFL0320w | 0 | |
| | | | PFL0840c | 1 | |
| | | | PFL1090w | 1 | Toxoplasma gliding-associated protein homolog45 |
| | | | PFL1110c | 1 | Cyclic nucleotide-binding domain |
| | | | PFL1165w | 0 | |
| | | | PFL1365w | 1 | |
| | | | PFL1435c | 1 | Myosin head motor domain ATPase (NOT non-specific coiled coil domain) |
| | | | PFL1515c | 0 | |
| | | | PFL1565c | 1 | |
| | | | PFL1700c | 1 | Vacuolar-type H+-translocating inorganic H+ pyrophosphatase |
| | | | PFL2215w | 1 | Actin |

| | | | | | |
|---|---|---|---|---|---|
| | | | PFL2225w | 1 | Calmodulin-like protein |
| | | | PFL2460w | 1 | Coronin-type WD-40 repeat |
| | | | PFL2510w | 1 | Chitinase |
