# Supplementary material for: Similarity searches in genome-wide numerical data sets
Source: Biol Direct. 2006 May 30;1:13. doi: 10.1186/1745-6150-1-13 (PMC1489924; doi:10.1186/1745-6150-1-13)
Supplement: Additional data file 4 — Figure, showing a) Factors likely regulating Poly(A)-tail synthesis and maturation, found by psi-square using Ptal as a query. Graph vertices are connected only if the corresponding proteins were found in the same purification. b) The intersection of three protein sets: 18 proteins found by psi-square when Ptal was used as a query; 20 proteins identified as factors regulating Poly(A)-tail synthesis and maturation by Gavin et al. (2002); 33 proteins found by psi-square with purification-made query. [file 1745-6150-1-13-S4.pdf]

a)

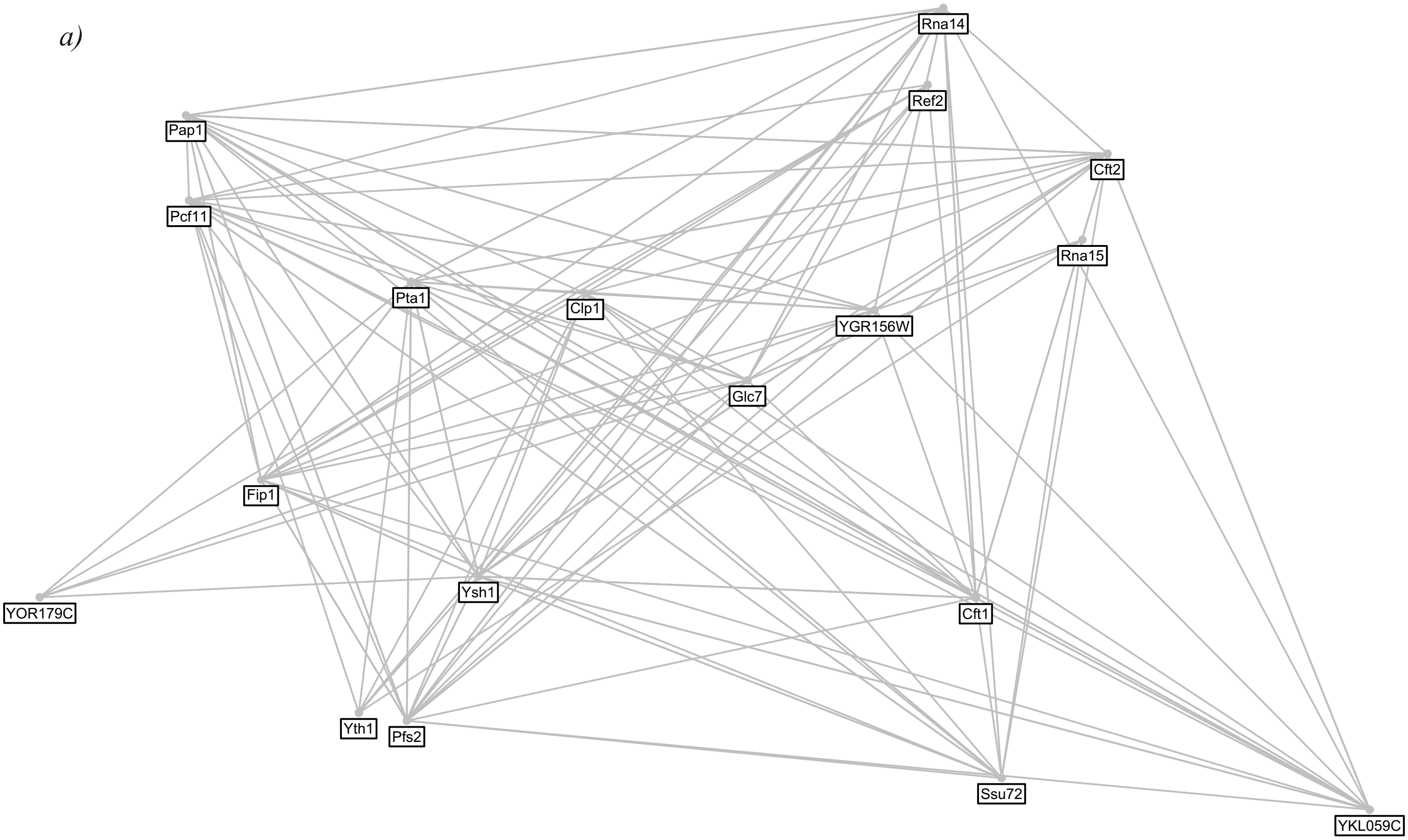

b)

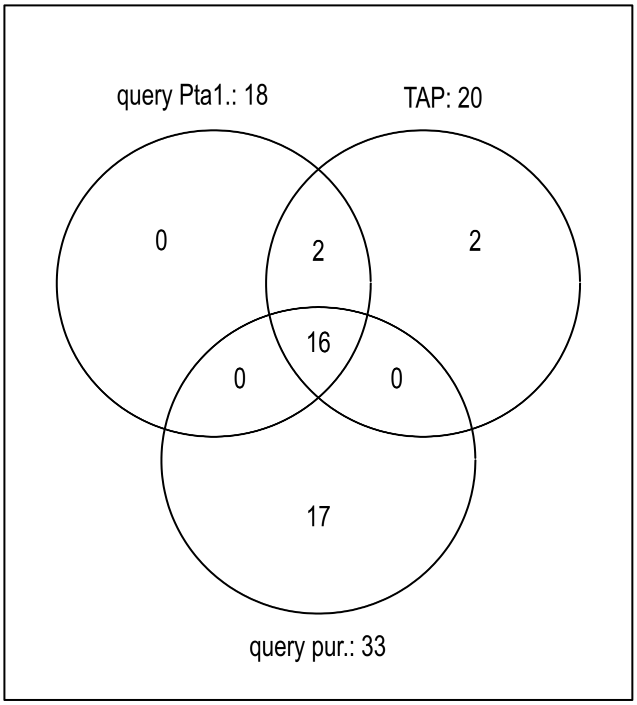

Figure. a) Factors likely regulating Poly(A)-tail synthesis and maturation, found by psi-square using Pta1 as a query. Graph vertices are connected only if the corresponding proteins were found in the same purification. b) The intersection of three protein sets: 18 proteins found by psi-square when Pta1 was used as a query; 20 proteins identified as factors regulating Poly(A)-tail synthesis and maturation by Gavin et al. (2002); 33 proteins found by psi-square with purification-made query.
