# Supplementary material for: Similarity searches in genome-wide numerical data sets
Source: Biol Direct. 2006 May 30;1:13. doi: 10.1186/1745-6150-1-13 (PMC1489924; doi:10.1186/1745-6150-1-13)
Supplement: Additional data file 5 — Figure showing factors likely regulating Poly(A)-tail synthesis and maturation, found by psi-square using purification as a query. [file 1745-6150-1-13-S5.pdf]

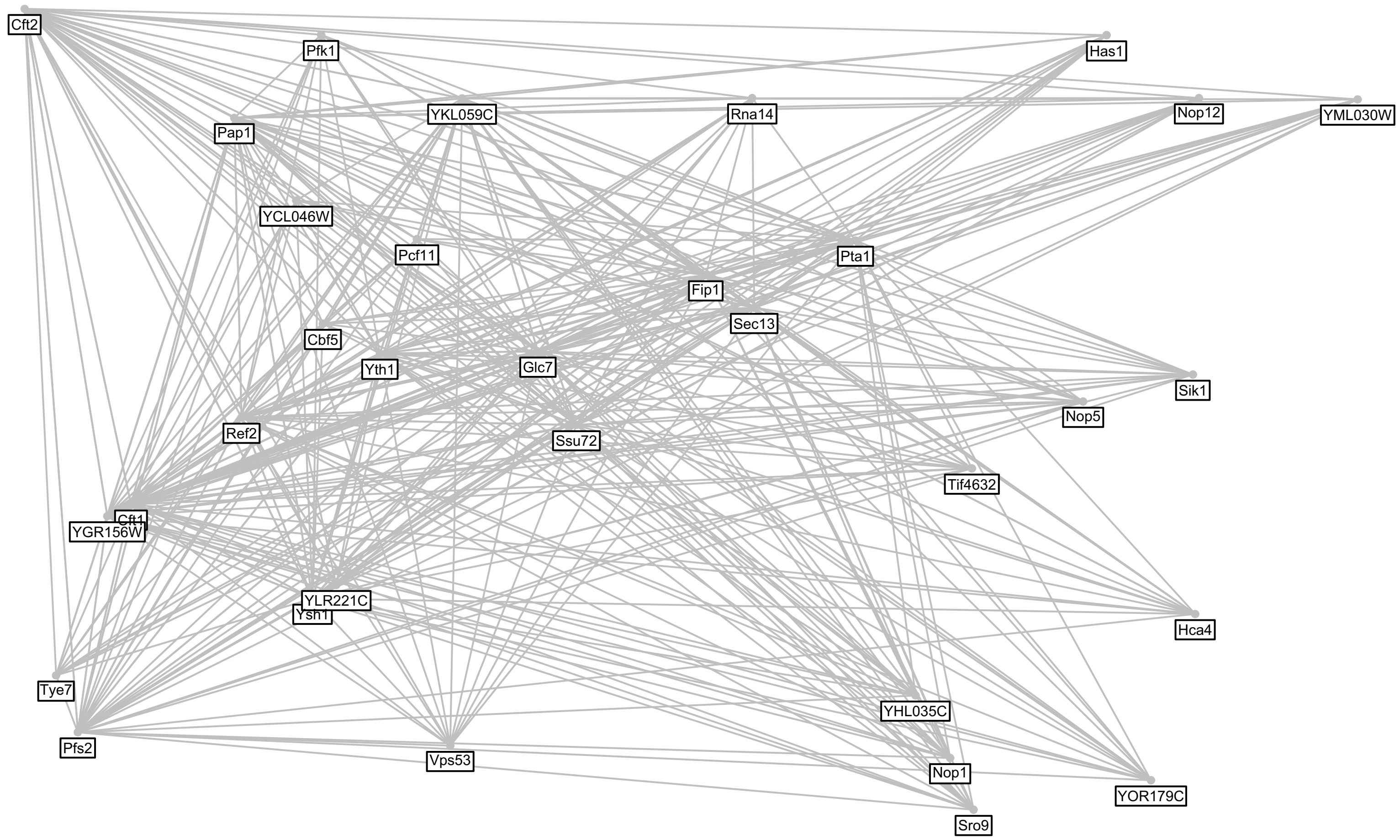

Figure. Factors likely regulating Poly(A)-tail synthesis and maturation, found by psi-square using purification as a query.
